# Content, Behavior Change Techniques, and Quality of Pregnancy Apps in Spain: Systematic Search on App Stores

**DOI:** 10.2196/27995

**Published:** 2021-11-17

**Authors:** Aranzazu Muñoz-Mancisidor, Ruben Martin-Payo, Xana Gonzalez-Mendez, María Del Mar Fernández-Álvarez

**Affiliations:** 1 Hospital Universitario San Agustin Avilés Spain; 2 Universidad de Oviedo Oviedo Spain; 3 Grupo de Investigación de Promoción de la Salud-Instituto de Investigación Sanitaria del Principado de Asturias Oviedo Spain

**Keywords:** pregnancy, mobile apps, behavior, technology assessment, biomedical, telemedicine

## Abstract

**Background:**

Women consult information in mobile apps (apps) during pregnancy, and even obstetrics specialists highlight that pregnancy is the ideal moment for the use of apps as consultation sources. However, the high number of apps designed for pregnancy requires a careful assessment to determine their suitability before recommendation.

**Objective:**

The aim of this study is to identify the apps available in Spanish that can be recommended based on their content, behavior change techniques (BCTs), and quality as a complementary tool during pregnancy.

**Methods:**

A systematic search on app stores to identify apps was performed in the Apple App Store and Google Play with the subject term “pregnancy.” The apps meeting the following criteria were chosen: pregnancy-related content, free, and available in Spanish. An app was excluded if it was classified as a game or entertainment and thus lacking an educational or health aim and if it did not target the population under study. The selected apps were downloaded, and their quality was assessed using the Mobile Application Rating Scale (MARS), with the BCTs included evaluated using the BCT taxonomy version 1 and its content.

**Results:**

A total of 457 apps were identified, 25 of which were downloaded for assessment (5.6%). The median for objective and subjective quality was 2.94 (IQR 2.71-3.46) and 1.75 (IQR 1.25-2.25), respectively. Regarding content, the median of topics included in the apps was 23 (IQR 16-23), with weight gain, nutrition, fetal development, and physical activity being the most common. The median number of BCTs was 12 (IQR 0.5-3.5). The most frequently identified BCTs in the apps were “Self-Monitoring of Outcomes,” followed by “Goal Behavior” and “Instructions.” Statistically significant correlations were observed between objective quality and content (ρ=0.624; *P*=.001), subjective quality and content (ρ=0.638; *P*=.001), objective quality and BCTs (ρ=0.672; *P*<.001), subjective quality and BCTs (ρ=0.623; *P*<.001), and BCTs and content (ρ=0.580; *P*=.002).

**Conclusions:**

The results of this study suggest that only a small percentage of free pregnancy apps available in Spanish should be recommended. The apps with the best MARS scores were those that addressed a higher number of topics and included a higher number of BCTs. Those with the best content and quality, and a higher number of BCTs included could be recommended by health professionals.

## Introduction

It is estimated there are more than 360,000 pregnancies each year in Spain, with the median age of pregnant women being approximately 32 years [1], which implies that there is a generation used to digital technology [2]. This characteristic, as well as the need to obtain information, can influence the use of mobile apps as a source of information [3]. Recent studies suggest that apps are more frequently consulted during pregnancy to look up different types of information, such as behavior or body changes [4].

Off-site health care, mediated by digital technology, has emerged in the past years in obstetrics and gynecology [5]. According to Greiner [6], for the use of this technology to be successful, it must address the values and interests of all parties involved in said use. For example, and specifically related to apps, pregnant women value very highly the inclusion of evidence-based information, experts’ opinions, and personalized tips in the app [4]. Therefore, the literature should highlight the importance of selecting appropriate behavioral change techniques (BCTs) in interventions with pregnant women, as not all interventions are equally effective [7]. There are several claims for the integration of apps as pregnancy-monitoring tools. For one, women usually respond quite positively to this integration, and they highlight the empowerment derived from these tools [3]. For another, some authors suggest that pregnancy is the best clinical time to use digital technology in terms of benefits [8]. Finally, there is an abundance of obstetrics and gynecology apps [9], and more specifically pregnancy apps [10].

This last aspect can also have a negative side that therefore must be considered. Carter et al’s [11] review highlights the benefits of apps in the support of decision-making during pregnancy, but it also highlights a lack of rigorous evaluation reports about the use and content of the apps. According to this, inappropriate app usage could harm the woman or the fetus.

This calls for a review and assessment of apps before recommendation, and while there are no specific criteria to unequivocally identify which elements in the app to assess [12], previous reviews of apps suggest assessing at least content, quality, and BCTs. There are several studies that have assessed these parameters in pregnancy apps in other languages [13,14], but no other study presenting the results of an assessment of apps available in Spanish has been found. Therefore, the objective of this study is to identify which apps available in Spanish can be recommended based on their content, quality, and BCTs as a complementary tool during pregnancy.

## Methods

### Study Design

This review used a step-by-step systematic approach that included 2 steps: (1) identifying and selecting the apps with the function of “pregnancy monitoring” available in the Apple App Store and Google Play (Android) between November 2020 and December 2020; and (2) assessing their quality, content, and BCTs.

### Step 1: Selection of Smartphone Apps

Our methods sought to replicate the way a patient might access a pregnancy app. Searches were performed in the Apple App Store and Google Play Stores using the word “pregnancy” in both stores. The searches were performed using an iPad Air (fourth generation; Apple Inc) and a Samsung Galaxy Tab A6 (Samsung Electronics Co, Ltd).

A first review of the apps based on their description in the digital stores was carried out. The apps meeting the following criteria were selected: content related to pregnancy, free, and available in Spanish. Apps were excluded if they were classified as a game or entertainment and therefore had no educational or health aim, or if they did not target the population under study (pregnant women).

The apps meeting the criteria specified above were downloaded, and a second review was carried out based on app usage. The same inclusion and exclusion criteria were used for the final selection, with malfunctioning or not working incorporated as exclusion criteria. The apps selected were labeled as recommended, and their quality, content, and BCTs were assessed.

### Step 2: Assessment of Smartphone Apps (Quality, Content, and Techniques)

#### Quality Assessment

The objective and subjective quality of each app was assessed by consensus between 2 researchers (RMP and MFA) using the Spanish version of the Mobile Application Rating Scale (MARS) [15]. This tool was chosen because of the good metric qualities of both its original English version and its Spanish adaptation (internal consistency α>.77; temporal stability *r*>0.72; interrater reliability >0.76) [16]. MARS has been validated for its use in health apps, and it has been used in several studies related to our research focus, such as specific nutrition apps for pregnancy [13,14,17]. MARS includes 23 items distributed in 2 subscales, objective quality (19 items distributed in 4 dimensions: engagement, functionality, esthetics, and information quality) and subjective quality (4 items), and 6 specific and independent items for health apps (awareness, improvement of knowledge, improvement of behaviors, change intention, social support, and behavior change). All items are rated on a 5-point scale (1, inadequate; 2, poor; 3, acceptable; 4, good; 5, excellent) with possible total scores being 1 to 5 for objective, subjective, and specific items. Mean scores were calculated for each domain (engagement, functionality, esthetics, and information), and overall app quality was calculated by averaging the aggregated mean for all domains [15].

#### Content Assessment

A content analysis strategy was developed by a researcher (AMM) with the aim of reviewing which pregnancy topics each app could address, and a thematic content analysis was developed. Finally, the content identified related to the target of our study was classified into categories.

#### Assessment of BCTs

The BCTs included were evaluated independently by 2 researchers (RMP and MFA). No major differences were observed between both researchers, and the final BCTs included were accorded by consensus. A behavioral change technique was only coded when there was clear evidence of its inclusion in the app. The behavioral change techniques used in each app were assessed using the BCT taxonomy version 1 (BCTTV1), which was originally developed by Michie et al [18] and has been shown to be a comprehensive, valid, and reliable approach for assessing techniques for changing behavior in pregnancy apps [7]. Scheoppe et al [19] and Martín-Payo et al [20] have applied a dichotomous scoring system to BCTs to indicate the absence (absence=0) or presence (presence=1) of each technique, permitting a total BCT score per app (possible score 0-93) to be generated.

### Data Analysis

Total scores, median, and IQR for each app on each domain of the MARS and the BCTTv1 were calculated. To determine if there was any relationship between app quality, BCTs, and content, Spearman’s rank correlation was used to determine any associations between MARS total scores, the number of topics, and BCTs. All statistical analyses were conducted using SPSS version 24.0 (IBM Corp) with significance levels set at a *P* value <.05.

## Results

**Figure 1:**
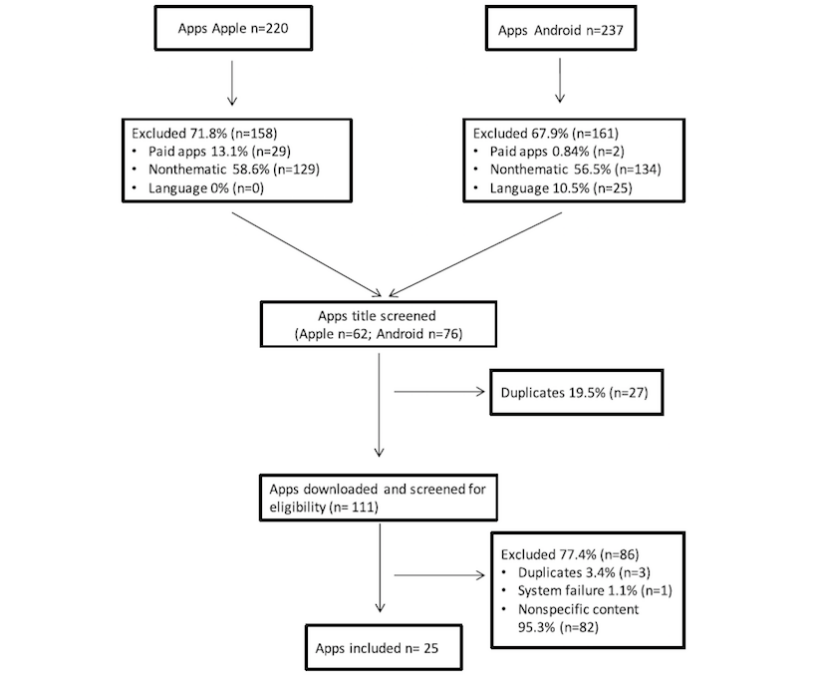
Flowchart of the app search process.

A total of 220 apps were identified in the Apple store, and 237 were identified in the Google (Android) app store; of these apps, 71.8% (n=158) and 67.9% (n=161) were excluded, respectively, for not meeting inclusion criteria. More specifically, 27 were duplicates, and 111 were downloaded, of which 77.4% (n=87) were excluded, with 25 (5.6%) apps retrieved for quality, content, and BCT assessment (Figure 1).

### Quality Assessment

The median in the different MARS dimensions was superior for objective quality than it was for subjective quality, with emphasis on functionality. The median for the specific part was lower than that for quality (Table 1).

**Table 1:** Mobile Application Rating Scale for app quality assessment (range 1 to 5).

Characteristic		Value, median (IQR)
**Objective quality**		2.94 (2.71-3.46)
	Engagement	2.60 (2.20-3.60)
	Functionality	4.00 (4.00-4.00)
	Esthetics	3.00 (2.67-3.67)
	Information	2.60 (2.00-3.00)
**Subjective quality**		1.75 (1.25-2.25)
	Would recommend	2.00 (1.00-2.00)
	Use after 12 months	2.00 (1.00-3.00)
	Payment required	1.00 (1.00-1.00)
	Rating	2.00 (1.00-3.00)
Awareness		2.00 (1.00-2.00)
Knowledge		2.00 (2.00-3.00)
Behavior		1.00 (1.00-3.00)
Change intention		2.00 (1.00-3.00)
Social support		2.00 (2.00-3.00)
Behavior change		1.00 (1.00-3.00)

### Content Assessment

A total of 28 topics were identified (Multimedia Appendix 1) with the median being 23 (IQR 16-26). The more frequent topics included in the apps were “weight gain,” “balanced diet,” “fetal development,” “physical exercise,” and “changes during pregnancy.” The lower number of topics included in an app was 11 and the highest was 28 (Table 2).

Positive and significant correlations were observed between the MARS scores and the total number of topics included (Table 3).

**Table 2:** Number of topics included in each app.

App	Topics included in the app, n
Seguidor de mi embarazo: Preglife	24
Mi embarazo semana a semana en español	25
Mi Embarazo día a día	21
Mi embarazo día a día: Semanas de embarazo español	25
Embarazadas primerizas	15
Guía para Embarazadas Primerizas Gratis	21
Embarazo Mes a Mes	18
Tu Embarazo Semana a Semana	25
Mi EMBARAZO por SEMANAS Calendario Maternidad	21
Embarazo semana a semana español días y meses	11
Embarazo semana a semana español	17
Cuidados en el Embarazo	11
Embarazo saludable	14
Mi embarazo como prepararse día a día	11
Embarazo +	27
Babycenter	27
iNatal	28
Embarazo Semana a Semana app	26
Embarazo. Sprout	24
Tu Embarazo	20
Mi embarazo Doctissimo	26
Yo Embarazo Ribera Salud	13
Gestavida	27
Embarazo Óptimo	28
Mi embarazo al día	23

**Table 3:** Correlation between Mobile Application Rating Scale scores and total topics included in the apps.

Characteristic	Correlation of total of topics included in the app, ρ	*P* value
Objective quality	0.624	.001
Subjective quality	0.638	.001
Awareness	0.537	.006
Knowledge	0.727	<.001
Behavior	0.539	.005
Change intention	0.565	.003
Social support	0.684	<.001
Behavior change	0.734	<.001

### BCT Assessment

A total of 12 different BCTs were identified, with a median of 2 (IQR 0.5-3.5).

The most frequently identified BCTs in the apps were “Self-Monitoring of Outcomes,” followed by “Goal Behavior” and “Instructions” (Table 4).

The Spearman correlation analysis showed a significant and direct association between the number of BCTs included in the app, their quality, and the number of topics addressed (Table 5).

**Table 4:** Percentage of apps that included each BCT

BCTs^a^	Apps that included each BCT, n (%) (N=25)
Self-monitoring of outcomes of behavior	11 (44)
Goal-setting behavior	10 (40)
Prompts/cues	9 (36)
Instruction on how to perform a behavior	9 (36)
Action planning	6 (24)
Goal setting outcome	6 (24)
Social support unspecified	5 (20)
Demonstration of the behavior	4 (16)
Self-monitoring of behavior	2 (8)
Credible source	1 (4)
Graded tasks	1 (4)
Monitoring of emotional consequences	1 (4))

^a^BCT: behavior change technique.

**Table 5:** Correlation between Mobile Application Rating Scale scores and total number of topics included in the apps with BCTs.

Characteristic	Correlation of number of BCTs^a^ included in the app, ρ	*P* value
Objective quality	0.672	<.001
Subjective quality	0.623	<.001
Awareness	0.510	.009
Knowledge	0.588	.002
Behavior	0.654	<.001
Change intention	0.572	.003
Social support	0.520	.008
Behavior change	0.668	<.001
Total number of topics included in the app	0.580	.002

^a^BCT: behavior change technique.

## Discussion

Although many hundreds of pregnancy apps are commercially available, of those retrieved in this study, only 25 contained potentially suitable pregnancy-specific content to be recommended to pregnant women. This means that, according to the criteria used by the researchers based on quality and content, approximately 5.5% (25/457) of the apps could be recommended. Previous studies have drawn similar conclusions and highlight that not all obstetrics-gynecology commercialized apps can be recommended [21,22] as similar percentages have been observed in other studies despite different selection criteria being used [23].

Although popular app ratings in some digital shops can be useful on some occasions, they are not free from manipulation, and market research suggests that more than half of the reviews on iOS for apps are fake [23]. For this reason, and considering the results obtained, we consider that health apps must be more rigorously evaluated.

There is a previous study in which the MARS was used to evaluate apps specifically designed for pregnancy in Australia [14]. Although the scores in our study cannot be directly compared with the ones in the Australian study, it can be confirmed that the quality of the apps evaluated was similar to the quality observed in our study. The use of the MARS provides important information about the app’s usability or user satisfaction, which is a side of apps usually ignored in health contexts but one that remains essential for the app’s feasibility and the effectiveness of its use. Other studies have found the users’ star rating to be an indicator of satisfaction, suggesting it as a predictor for app download and usage [24]. In general, the majority of the apps seem to work properly according to the objective quality scores (engagement, functionality, esthetics, and information) but not according to subjective quality scores. This may be related to the characteristics of the items that compose the app. For example, “using the app after 12 months” does not apply considering the planned used of the app, as pregnancy is shorter. On the other hand, the dislike for the “pay for the use of the app,” which obtained the lowest score, can be attributed to the characteristics of the Spanish health system, as a great part of the population perceives health care as free, and therefore, any payment related to health services is not popular.

The most common contents in the majority of apps were related to “weight gain,” “nutrition,” “fetal development,” “physical activity,” and “changes during pregnancy,” and they tended to include self-monitoring and goal-setting behaviors. The inclusion of these topics in the apps can be potentially considered as very effective. Adoption of healthy behaviors during pregnancy can potentially improve maternal and child health. Adverse perinatal health outcomes are associated with maternal risk factors that may be modifiable through changes in maternal behavior [25,26]. Previous studies show that the use of apps has been effective in the improvement of women’s knowledge or even in the promotion of healthy behaviors such as physical activity [27] or healthy eating [28]. Overdijkink et al [29] reached the same conclusion and highlighted the positive influence of using apps for gestational weight gain and increased vegetable and fruit intake, among others. Being able to recommend trustworthy apps as pregnancy tools may contribute to helping those population groups previously described by some authors as “at risk” due to their difficulties in adhering to behavioral recommendations [30].

This study discovered that generally a limited number of BCTs are used in apps specifically designed for pregnancy. The number of BCTs identified is consistent with the number identified by Brown et al [13], and it is superior to the number identified by Musgrave et al [14], at 11 and 5 BCTs, respectively. The consistency of results is not limited to the number of BCTs but also applies to the BCTs included despite the use of different taxonomies. As some authors suggest, the inclusion of BCTs contributes to improving the potential to promote behavior change [31]. This probably justifies the employment of different BCTs in digital behavior change interventions [32]. In this sense, Webb et al [33] conclude that the inclusion of BCTs is linked to the efficacy of interventions in which digital resources are used, a possible motivator for the potential incorporation of more specific BCTs by app developers. From a behavioral point of view, it makes sense to include more appropriate BCTs depending on the objectives [31]. However, it has been observed that a considerable number of apps do not include BCTs. In the literature consulted, these apps do not seem to be the more effective, and thus performing more analytical studies to prove this hypothesis appears warranted.

The results of this study are therefore extremely useful for clinical practice. As presented, apps specifically designed for pregnancy can be very positively evaluated by health professionals and more specifically, by midwives, who monitor low-risk pregnancies in the Spanish health system. Therefore, while health professionals are essential for appropriate pregnancy monitoring [34], apps can be used as complementary care.

Finally, although different criteria could be used to assess and evaluate the eligibility of the apps to be recommended [12], the choice of content selection, MARS scores, and BCTs seem to be adequate as a correlation has been observed among the 3 elements. This could suggest that a ranking could be established for app recommendation, with those apps with better quality, content, and BCTs scoring at the top.

Some limitations of this study should be noted. Our search was restricted to free apps. This was deliberate because we did not want to include those that would incur a cost to people. Another possible limitation is related to the search strategy used. The lack of standardized search terms may lead to the use of those apps considered more adequate by the researchers according to their own experience. Considering the dynamism of the app market, it is possible that future searches will identify different apps, and therefore some cannot be available for recommendation. Finally, no previous research assessing the effectiveness of apps could be found. Future research is needed for assessment because health professionals might not prescribe health apps due to distrust and a lack of knowledge about their efficacy [35].

The results of this study suggest that only a small percentage of free pregnancy apps available in Spanish should be recommended. The apps with the best MARS scores were those that addressed a higher number of topics and included a higher number of BCTs.

Those with best content, quality, and a higher number of BCTs included could be recommended by health professionals.

## Appendix

Multimedia Appendix 1 Mobile Application Rating Scale and behavioral change techniques included in the apps.

## Abbreviations

BCT: behavior change technique
BCTTV1: behavior change technique taxonomy version 1
MARS: Mobile Application Rating Scale
